# Implementing effective eLearning for scaling up global capacity building: findings from the malnutrition elearning course evaluation in Ghana

**DOI:** 10.1080/16549716.2020.1831794

**Published:** 2020-10-22

**Authors:** Reginald Adjetey Annan, Linda Nana Esi Aduku, Samuel Kyei-Boateng, Ho Ming Yuen, Trevor Pickup, Andy Pulman, Michele Monroy-Valle, Ann Ashworth, Alan A. Jackson, Sunhea Choi

**Affiliations:** a Department of Biochemistry and Biotechnology, Kwame Nkrumah University of Science and Technology (KNUST), PMB University Post Office, Kumasi, Ghana; b Faculty of Medicine, University of Southampton, Level B, South Academic Block, Southampton General Hospital, Southampton, UK; c Facultad de Ciencias de la Salud, Universidad Rafael Landívar, Ciudad de Guatemala, Guatemala; d Department of Population Health, London School of Hygiene and Tropical Medicine, London, UK; e Human Development and Health, Faculty of Medicine, University of Southampton, Southampton, UK

**Keywords:** Global capacity building, challenges and opportunities, severe acute malnutrition, eLearning delivery

## Abstract

**Background:**

Global demand for capacity building has increased interest for eLearning. As eLearning resources become more common, effective implementation is required to scale up utilization in Low- and Middle-Income Countries (LMICs).

**Objective:**

This paper describes the process of implementing a malnutrition eLearning course, effectiveness of course delivery models devised, factors affecting course completion, and cost comparison between the models and face-to-face training at healthcare and academic institutions in Ghana.

**Methods:**

Four delivery models: Mobile Training Centre (MTC), Online Delivery (OD), Institutional Computer Workstation (ICW) and Mixed Delivery (MD) – a combination of OD and ICW – were determined. Participants were enabled to access the course using one of the four models where contextually appropriate. Pre and post-assessments and questionnaires were administered to compare participants’ course completion status and knowledge gain between delivery models. The effect of access to computer and Internet at home and relevance of course to job and academic progression on course completion were further investigated. Comparison of delivery model costs against face-to-face training was also undertaken.

**Results:**

Of 7 academic and 9 healthcare institutions involving 915 people, 9 used MTC (34.8%), 3 OD (18.8%), 3 ICW (34.2%) and 1 MD (12.2%). Course completion was higher among institutions where the course was relevant to job or implemented as part of required curriculum activities. Knowledge gain was significant among most participants, but higher among those who found the course relevant to job or academic progression. The implementation costs per participant for training with MTC were £51.0, OD £2.2, ICW £1.2 and MD £1.1, compared with a face-to-face training estimate of £105.0 (1 GHS = 0.14 GBP).

**Conclusion:**

The malnutrition eLearning course makes global capacity building in malnutrition management achievable. Adopting contextually appropriate delivery models and ensuring training is relevant to job/academic progression can enhance eLearning effectiveness in LMICs.

## Background

The global demand for capacity building has increased interest in, and demand for, non-traditional training modalities, such as eLearning, to increase training coverage and to improve the efficiency of training delivery [1]. This demand, coupled with improved access to internet globally and the fast-advancing digital technologies, has increased the development of technology-enhanced/enabled learning/training interventions, also known as eLearning [2,3]. As such interventions become more available, it is important to explore their effective implementation and utilization to ensure optimal benefits [4]. Durlak and DuPre, in their systematic review of over 500 prevention and health promotion programmes, reported that implementation factors affected outcomes [5]. Making eLearning interventions available to target users does not of itself ensure they will make use of them. There is therefore a great need to support effective implementation of available eLearning interventions to enable global capacity building [6,7].

An eLearning intervention is a means to achieve a goal [8]. Such a goal could be to deliver a curriculum, facilitate flexible learning, widen access to learning/training opportunities or offer continuous professional development (CPD) and in-service training. In order for the goal to be achieved, the eLearning intervention must be implemented appropriately to help realize its intended benefit. Factors, which have been identified to make eLearning implementation and utilization successful, can be summarized into four broad categories [8]. The first is infrastructural support, including hardware, software and information technology (IT) support. In the LMIC setting this also includes availability of electricity. The second is institutional capability and resources, including financial and human, availability of trainers/educators and institutional reorientation from face-to-face to eLearning. The third category is an inbuilt capability to follow up and measure success of eLearning as a means of training. This includes eLearning utilization and measurable achieved outcomes or impacts purposed by the eLearning. The fourth is recognition for eLearning, which involves eLearning integration into curricula and/or recognition as CPD [9,10].

Recent analysis of trends reveals significant reductions in malnutrition globally. But this is not the case in many countries in sub-Saharan Africa (SSA) where the magnitude of wasted children remains unchanged [11]. In 2019, there were 47 million wasted (acutely malnourished) children globally, of which 14.3 million were severely wasted [12]. The SSA region accounted for 23% of the global wasting burden in 2016 [13]. In Ghana, the prevalence of moderate or severe wasting was 9% in 2008, 6% in 2011 and 4.7% in 2014, according to the Demographic and Health Survey [14]. SSA as a region is not on course towards achieving a wasting target of less than 5% by 2025 which was set by the World Health Assembly [13] and Covid-19 is expected to worsen the situation as it is adversely affecting food security [15].

Appropriate management of acutely malnourished children is critical for child survival as these children face immediate risk of death if not properly managed [16]. The World Health Organisation (WHO) has provided guidelines on the management of children with severe acute malnutrition (SAM) – more commonly known as WHO ‘Ten steps’ for management of SAM [17]. The guidelines are effective in treating severely malnourished children and, if scaled up, are expected to reduce deaths substantially [18–21]. The management of SAM is among the list of effective nutrition interventions [21] and is considered a key component of the United Nation’s Scaling Up Nutrition (SUN) framework. The challenge is how to ensure appropriate management of these children, since it requires effective training/capacity building interventions and implementation of these interventions at scale [22].

In countries most affected by SAM, lack of operational capacity for scaling up SAM management has been reported [23,24]. Training and curricula are outdated, non-existent [25] or misaligned with national guidelines, resulting in healthcare systems with a workforce unable to effectively identify and treat malnourished children [26]. There have been several global calls to make the identification and treatment of SAM a core competency for health professionals [27,28] and appeals for global scale-up of SAM management. These are important for achieving Sustainable Development Goal number 2 [29].

In response to these calls, the University of Southampton and International Malnutrition Task Force of the International Union of Nutritional Sciences jointly developed an eLearning course, called ‘Caring for infants and children with severe acute malnutrition’ [30,31]. The course has 3 modules with each taking 2–3 hours to complete. The course is based on the WHO guidelines for the management of SAM and provides learning on: how to differentiate between chronic and acute malnutrition; pathophysiological changes in malnutrition and consequences for treatment; how to assess and screen children for malnutrition and interpret the results for action; how to manage malnourished children using the WHO Ten Steps; hospital compared with community management; the importance of an integrated approach between hospital and community; and how to support mothers and carers to prevent the recurrence of malnutrition. The course is interactive, scenario-based and designed around a hypothetical nutrition community in which children and their care are the focus for learning. The content structure and design support apprehending structure, integration, application and reflection. The content is set at a level suitable for in-service and pre-service health professionals who are, or will be, working with undernourished children. Further details are described elsewhere [30–33]. Following the development of the malnutrition eLearning course, various dissemination approaches, including institutional grants and best practice awards for curriculum implementation, social media campaigns, exhibitions and regular release of newsletters, were used to encourage and scale up its utilization. These activities led to over 16,000 people across 120 countries using the course by 2016 [30].

With support from the UK Department for International Development, as part of the Nutrition Embedding Evaluation Programme, an impact evaluation study was carried out from 2014 to 2017. This investigated firstly whether the malnutrition eLearning course improved knowledge and skills of in-service and pre-service health professionals in managing children with SAM and enabled them to apply their gained knowledge and skills in patient care; secondly, if it led to improved diagnosis, clinical management and survival of children with SAM. The study showed that the course improved knowledge, understanding and skills of health professionals in the diagnosis and management of children with SAM, and led to changes in clinical practice and improved confidence [32]. The course also led to improvement in the identification of SAM, improvements in almost all aspects of the WHO ‘Ten Steps’ of case-management, and a reduction in case-fatality [33].

This paper describes the process of implementing the malnutrition eLearning course in Ghana, effectiveness of delivery models devised, factors that affected course completion and benefits gained from the course, and cost comparison between the delivery models implemented at study institutions and face-to-face training. Findings from the study provide opportunities for scaling up capacity building for malnutrition management and other health-related training in LMICs.

## Methods

The malnutrition eLearning evaluation (MeLE) was a prospective, longitudinal, cross-country, interrupted time-series study that took place in Ghana, Guatemala, El Salvador and Colombia. The outcomes and impact of the malnutrition eLearning course on knowledge gain, changes in practice and clinical outcomes were measured and details of the methods and findings have been published [32,33]. As part of the study, we devised, implemented and evaluated different delivery models to overcome contextual IT infrastructure challenges in Ghana. In this paper we report the process evaluation and associated findings.

## Participants

Students of health training academic institutions (pre-service) and healthcare professionals (in-service) were the target study participants. Of 9 academic and 10 healthcare institutions invited, 7 and 9 participated in the study, respectively. Academic institutions that participated were from 3 universities across Ghana, 3 nursing and midwifery training colleges in the Ashanti Region and 1 Health Training College. The healthcare institutions that participated were 1 regional hospital, 7 district hospitals and 1 specialised children’s hospital and their linked health community centres. Full details of the study participants are reported elsewhere [32,33].

## Research questions

The research questions investigated for this part of the study were: 1) what factors affected completion of the malnutrition eLearning course; 2) how did the different delivery models, relevance to job and curriculum integration affect course completion and knowledge gain; and 3) what were the implementation costs of the different models compared with face-to-face training delivery.

### Different delivery models design and implementation

A four-step process was used for designing and implementing different delivery models for the malnutrition eLearning course (see Supplementary file 1).

#### Step 1. Identifying factors that would influence course implementation in the study contexts

A preliminary investigation was conducted to determine key factors that were to be addressed at participating institutions. This was done through literature review, institutional surveys and visits to participating institutions. Factors that influence eLearning implementation were identified through literature review in four categories: 1) IT infrastructure; 2) institutional capability; 3) inbuilt capability for assessment; and 4) recognition. Next, surveys and visits to participating institutions were conducted to assess these factors at each institution. Teaching on malnutrition and its management in the health science programs at participating academic institutions and in-service training available at healthcare institutions, and institutional capability to provide teaching/training on malnutrition and its management were assessed through surveys. The research team visited participating institutions to assess IT infrastructure for eLearning delivery, including availability of computer workstations with working computers and Internet connectivity. The likelihood of individual participants having access to computer and Internet at home was considered. Discussions with staff managing the workstations were held to determine challenges for eLearning delivery relevant to their institution.

#### Step 2. Determining solutions to address the challenging factors identified in Step 1

The findings from step 1 suggested that we needed to address 1) IT infrastructure to deliver the course at the participating institutions and 2) integration of the course into curricula. For these we developed two mitigating solutions: a) different delivery models to overcome IT infrastructure challenges and b) a capacity building workshop for educators/trainers to facilitate curriculum review and integration of the malnutrition course into curricula and its effective utilization for curriculum/in-training delivery. The workshop was implemented and evaluated, details of which will be reported separately.

#### Step 3. Devising and mapping suitable delivery models

Four delivery models were devised to suit different institutional contexts: Online Delivery (OD), Institutional Computer Workstation (ICW), Mobile Training Centre (MTC) and Mixed Delivery (MD) – Supplement file 2. Where computers and internet access were available, the online course was introduced – OD. If an institution had a workstation with computers but no or poor internet connectivity, an offline version of the course was installed onto workstation computers for participants to use – ICW. For institutions with no workstations or internet connectivity and some individuals having access to personal computers, a temporary training facility was devised – MTC. This involved gathering participants at one location where laptop computers were provided by the research team with the course pre-installed onto them. Those who had personal computers were asked to bring their own computers and the course was installed onto their computers. If an institution had workstations but were limited in capacity, we used a mixed model of workstation and individual computers – MD. This involved making an offline version of the course available on participants’ computers and introducing the online course.

#### Step 4. Implementing the different delivery models

The research team and each participating institution agreed on the most suitable delivery model for the course. Each academic institution decided how and where to integrate the course into their health science curriculum, and whether to consider it as additional or assessed learning. Healthcare institutions identified the staff whose job responsibilities included caring for malnourished children and who would benefit from the training. Study participation was voluntary. This was followed by testing access to the online course from institutional workstation computers or, if needed, installing the offline course onto workstation computers. For MTC and MD, it was agreed that the research team would install the offline course during the course introduction session.

On the agreed date the research team gave a one hour introduction session and afterwards the participants in the MTC group were given 2–3 day scheduled, self-directed, training sessions and those in OD, ICW and MD groups were given 3 weeks to complete the course. Two institutions in OD, ICW and MD groups, which integrated the eLearning course as their required curriculum activities, timetabled self-directed learning sessions, and the other institutions recommended their students to take the course in their own time.

### Data collection

Pre and post-assessments and questionnaires were administered. Two sets of comparable assessments were prepared to measure knowledge gain from the course (see Supplementary file 3). Each assessment consisted of 32 questions on key topics identified from the course, and the questions were prepared to assess comprehension, application and integration of knowledge. Participants ranged from medical doctors to students (pre-service) and community health workers, and differences in existing knowledge between professional groups as well as within each group were anticipated. Further details of assessment setting are described elsewhere [32]. The pre-questionnaire was used to collect data on participants’ access to computer and Internet at home, and the post-questionnaire asked about course completion.

### Course completion

The post-questionnaire included a question about the extent to which participants completed the course and this was categorized as Completed, In progress or Not completed. The data were analyzed descriptively by institution. The effect of the different delivery models, participants’ access to computers and Internet at home and relevance of the course to job/academic progression on course completion was investigated.

We considered the participants from the healthcare institutions homogeneous and treated them as a single group. This was based on the participants’ reason for attending the training – ‘relevant to their job’ and also because they chose to participate in the training. Academic institutions were categorized into 2 groups based on relevance of the course to their academic progression. Two academic institutions integrated the course into their curricula as required/assessed learning. We grouped these participants with those from the healthcare institutions into ‘Relevant to job/academic progression’. Three academic institutions introduced the course as additional learning, and these participants were classified as ‘Not relevant to job/academic progression’. Chi-square tests were performed to investigate the relationships between the extent of course completion and the relevance of the course to job/academic progression and access to computers and Internet at home.

### Knowledge gain

The relationships between the extent of course completion, type of delivery model and relevance of the course to job/academic progression on knowledge gained from the course were investigated. Excel macros were prepared to automate the marking of assessments. Questions with no answers were treated as incorrect answers. Descriptive statistics were used to summarize the pre and post-assessment scores. Paired *t* tests were performed to assess the pre and post-assessment scores 1) by course completion and institution, and 2) by course completion and relevance to job/academic progression. Independent samples *t* tests were performed comparing the post-pre assessment differences between the relevance to job/academic progress subgroups by course completion. Mean difference with 95% CIs were presented. Statistical significance was set at the 5% level.

### Determination of implementation costs for the different delivery models vs. face-to-face training

To determine the cost for course delivery, itemized, unit and total costs spent for the execution of the different models were calculated. Costs included in the calculation were limited to those directly relevant for course delivery: trainers/facilitators, transportation of trainers/facilitators and trainees/participants, refreshment and hiring of training venue. The same analysis was done for delivering an equivalent face-to-face training on SAM management. Costs for equipment and development of face-to-face training and the malnutrition eLearning course were excluded from the analysis. This is because facility setup and intervention development costs are course-specific (for example customized vs template-based, fidelity of technology and level of interactivity used) and are made at one point in time. Supplementary file 4 explains the malnutrition eLearning development cost in context.

## Results

### Delivery models mapped and implemented

Supplementary file 2 shows the process of mapping 4 delivery models to 16 participating institutions based on their IT infrastructure resulted in 9 healthcare institutions adopting MTC, while in 7 academic institutions, 3 adopting OD, 3 ICW, and 1 MD.

### Participants and course delivery

Table 1 summarizes the institutions and individuals who participated in MeLE. In Ghana, 931 pre-service and in-service health professionals participated in the self-directed training with the malnutrition eLearning course. Of 915 who consented to MeLE, about a third of the participants accessed the course through MTC (318, 34.8%), OD (172, 18.8%), ICW (313, 34.2%) and MD (112, 12.2%). Of the 548 who participated in the post study, 291 were through MTC, 118 OD, 54 ICW and 85 MD. Two academic institutions were unable to facilitate access to the course during the study period and missed the post-study data collection. They were excluded from the analysis.

**Table 1.** Participating institutions, course delivery models and individual participants

Institution		Self-directed training				MeLE study		
Type	Name	Delivery model^d^		Training organization^e^	Participated in training	Consent for study	Participated in pre-study	Participated in post-study
Healthcare^a^	MCHH	MTC		Scheduled	33	31	31	27
	SMiH	MTC		Scheduled	38	38	38	34
	EGH	MTC		Scheduled	43	43	42	41
	APH	MTC		Scheduled	37	37	37	34
	SPH	MTC		Scheduled	34	33	32	30
	KGH	MTC		Scheduled	30	30	30	28
	SMaH	MTC		Scheduled	38	35	35	33
	KSH	MTC		Scheduled	40	40	40	37
	MGH	MTC		Scheduled	37	31	30	27
**Sub-total**					**330**	**318**	**315**	**291**
Academic^b^	KNUST		OD	Scheduled	36	36	36	35
	CHK		OD	Scheduled	75	71	71	68
	UHAS		OD	Independent	65	65	65	15
	F-CHNTS		ICW	Independent	58	58	58	54
	KNTC^c^		ICW	Independent	100	100	100	-
	NMTCK^c^		ICW	Independent	155	155	155	-
	CSUC		MD	Independent	112	112	108	85
**Sub-total**					**601**	**597**	**593**	**257**
**Total**					**931**	**915**	**908**	**548**

^a^Nine hospitals and their linked community centres. MCHH: Maternal and Child Health Hospital, SMiH: St Michael’s Hospital, EGH: Ejura Government Hospital, APH: Agogo Presbyterian Hospital, SPH: St Patrick’s Hospital, KGH: Kogongo Government Hospital, SMaH: St Martin’s Hospital, KSH, Kumasi South Hospital, MGH: Mankranso Government Hospital.

^b^Three universities and 4 health and nursing/midwifery training colleges. KNUST: Kwame Nkrumah University of Science and Technology, CHK: College of Health Kintampo, UHAS: University of Health and Allied Sciences, F-CHNTS: Community Health Nurses Training School – Fomena, KNTC: Kokofu Nurses’ Training College, NMTCK: Nursing and Midwifery Training College Kumasi, CSUC: Christian Services University College.

^c^Did not participate in the post-study and excluded from the analysis.

^d^Delivery models. MTC: Mobile Training Centre, OD: Online Delivery, ICW: Institutional Computer Workstation and MD: Mixed Delivery.

^e^Scheduling of self-directed training: scheduled – self-directed training sessions were organized by institutions, independent – participants were recommended to take the eLearning course on their own time.

### Course completion

Table 2 summarizes the course completion status reported by participants post-training in relation to delivery models and relevance of the course to job/academic progression. Overall, 354 (66.0%) reported to have completed the course, 100 (18.7%) reported they were in progress with the course and 82 (15.3%) stated they did not complete it. The percentage of participants completing the course at each institution varied widely, ranging from 100% to 25.3%.

**Table 2.** Course completion in relation to delivery models and relevance to job/academic progression

	Healthcare^a^	Academic^b^				
Institution	9 Hospitals	KNUST^c^	CHK^c^	UHAS^d^	F-CHNTS^d^	CSUC^d^
Delivery model^e^	MTC	OD	OD	OD	ICW	MD
Relevance to job/academic progression^f^	Yes	Yes	Yes	No	No	No
**Post-study course completion, N (%)**						
Total N	291	35	68	15	54	85
*Completed, N (%)*	215 (75.2%)	27 (79.4%)	67 (100%)	4 (26.7%)	20 (39.2%)	21 (25.3%)
*In progress, N (%)*	39 (13.6%)	3 (8.8%)	0 (0.0%)	8 (53.3%)	10 (19.6%)	40 (48.2%)
*Not completed, N (%)*	32 (11.2%)	4 (11.8%)	0 (0.0%)	3 (20.0%)	21 (41.2%)	22 (26.5%)
*Missing, N*	5	1	1	0	3	2

^a^Nine hospitals and their linked community centres. MCHH: Maternal and Child Health Hospital, SMiH: St Michael’s Hospital, EGH: Ejura Government Hospital, APH: Agogo Presbyterian Hospital, SPH: St Patrick’s Hospital, KGH: Kogongo Government Hospital, SMaH: St Martin’s Hospital, KSH, Kumasi South Hospital, MGH: Mankranso Government Hospital.

^b^Three universities and 2 health and nursing/midwifery training colleges. KNUST: Kwame Nkrumah University of Science and Technology, CHK: College of Health Kintampo, UHAS: University of Health and Allied Sciences, F-CHNTS: Community Health Nurses Training School – Fomena, CSUC: Christian Services University College.

^c^The eLearning course formed part of formal curriculum activities and timetabled in the MeLE study period.

^d^The eLearning course was introduced as an additional learning resource in the MeLE study period.

^e^Delivery models. MTC: Mobile Training Centre, OD: Online Delivery, ICW: Institutional Computer Workstation and MD: Mixed Delivery.

^f^The eLearning course content was relevant to participants’ jobs (in-service) or assessed for academic progression (pre-service).

#### Delivery models and course completion

The course completion rates in MTC (75.2%) and OD (83.0%) groups were higher than ICW (39.2%) and MD (25.3%); however, a subgroup analysis revealed a variation within the OD group with the course completion at one institution being 26.7%, which was similar to ICW and MD groups.

#### Relevance to job/academic progression and course completion

The course completion rates at the healthcare institutions (75.2%), and at KNUST (79.4%) and CHK (100%) where the course was implemented as part of their required curricula activities, were much higher than the three other academic institutions (UHAS, F-CHNTS and CSUC) where the course was introduced as additional learning in the MeLE study period, ranging from 25.3% to 39.2%.

#### Access to computer/Internet at home and course completion

Course completion was not associated with access to computer (*P* = 0.398) or Internet at home (*P* = 0.134) (Supplementary file 5).

#### Access to computer/Internet at home, relevance to job/academic progression and course completion

**Figure 1.**
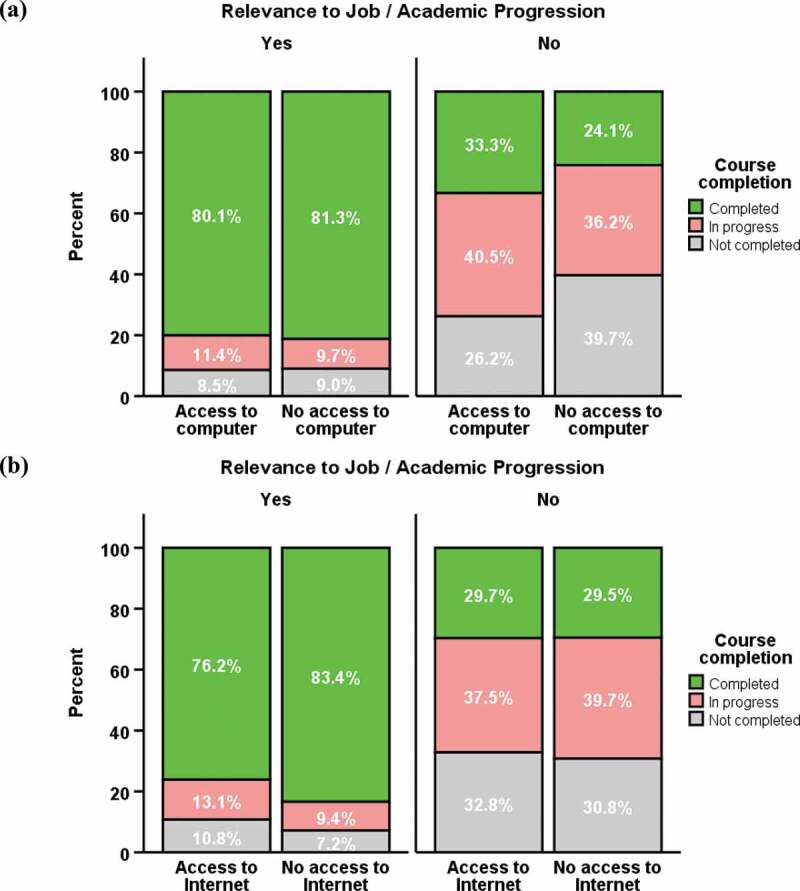
(a) Course completion and access to computer at home – no statistically significant association was found in the ‘relevant to job/academic progression’ group (*P* = 0.880) and ‘not relevant to job/academic progression’ group (*P* = 0.212); (b) Course completion and access to Internet at home – no statistically significant association was found in the ‘relevant to job/academic progression’ group (*P* = 0.246) and ‘not relevant to job/academic progression’ group (*P* = 0.955)

Regardless of having access to computer at home, the course completion rate was almost 3 times higher in the ‘relevant to job/academic progression’ group than those in the ‘not relevant’ group (Figure 1(a)). Similar results were found regarding access to Internet at home (Figure 1(b)).

Course completion rate of the ‘not relevant to job/academic progression’ group was significantly lower than the ‘relevant to job/academic progression’ group irrespective of home access to computer (33.3% vs 80.1%) or not (24.1% vs 81.3%), and Internet (29.7% vs 76.2%) or not (29.5% vs 83.4%), *P* < 0.001 (Supplementary file 6).

### Knowledge gain

Overall, most institutions showed a significant knowledge gain from the course (difference between pre and post-assessments), ranging from 4.4% to 23.1% (Table 3). However, the mean score of the post-assessment from one institution, UHAS, was lower than the pre-assessment.

**Table 3.** Course delivery models, relevance to job/academic progression, course completion and knowledge gain

		Healthcare^a^	Academic^b^				
Institution		9 Hospitals	KNUST^c^	CHK^c^	UHAS^d^	F-CHNTS^d^	CSUC^d^
Delivery model^e^		MTC	OD	OD	OD	ICW	MD
Relevance to job/academic progression		Yes	Yes	Yes	No	No	No
**Pre and post-assessments: mean percentage (SD)**							
Overall	*N*	264	34	67	14^g^	51	64
	*Pre*	26.8 (10.0)	24.6 (9.8)	33.4 (7.7)	36.4 (11.8)	23.9 (6.6)	23.6 (8.3)
	*Post*	42.6 (12.1)	47.8 (13.1)	48.3 (8.9)	34.6 (15.0)	28.4 (6.9)	28.2 (10.4)
	*Diff (Post – Pre)*	15.8 (11.7)	23.1 (14.6)	14.8 (9.9)	−1.8 (9.9)	4.4 (9.2)	4.6 (9.4)
	*95% CI*	(14.3, 17.2)	(18.0, 28.3)	(12.4, 17.2)	(−7.5, 3.9)	(1.9, 7.0)	(2.2, 6.9)
	*P*^g^	<0.001	<0.001	<0.001	0.502	0.001	<0.001
Completed	*N*	197	27	67	4^g^	20	16
	*Pre*	26.6 (9.8)	26.0 (10.1)	33.4 (7.7)	41.3 (8.4)	25.2 (6.5)	26.7 (7.5)
	*Post*	41.9 (12.2)	50.5 (12.0)	48.3 (8.9)	39.1 (14.4)	31.3 (5.8)	36.5 (10.0)
	*Diff (Post – Pre)*	15.4 (11.7)	24.5 (14.2)	14.8 (9.9)	−2.3 (13.1)	6.1 (9.2)	9.8 (10.1)
	*95% CI*	(13.7, 17.0)	(18.9, 30.1)	(12.4, 17.2)	(−23.2, 18.6)	(1.8, 10.4)	(4.5, 15.2)
	*P*^f^	<0.001	<0.001	<0.001	0.751	0.007	0.001
In progress	*N*	36	3	0	7	10	26
	*Pre*	31.7 (11.6)	18.2 (7.8)	-	34.3 (13.7)	21.5 (6.5)	24.2 (9.0)
	*Post*	46.6 (13.9)	44.8 (16.5)	-	34.7 (16.6)	25.8 (8.9)	27.3 (9.4)
	*Diff (Post – Pre)*	14.9 (13.1)	26.6 (21.8)	-	0.4 (10.4)	4.4 (10.9)	3.1 (9.3)
	*95% CI*	(10.5, 19.3)	(−27.5, 80.7)	-	(−9.2, 10.0)	(−3.4, 12.2)	(−0.7, 6.8)
	*P*^f^	<0.001	0.169	-	0.926	0.237	0.103
Not completed	*N*	31	4	0	3	21	22
	*Pre*	22.9 (6.1)	20.4 (8.1)	-	34.8 (13.2)	23.9 (6.8)	20.6 (7.2)
	*Post*	42.2 (8.2)	31.9 (6.1)	-	28.4 (15.3)	26.8 (5.9)	23.2 (8.2)
	*Diff (Post – Pre)*	19.3 (9.6)	11.5 (9.7)	-	−6.4 (2.8)	2.9 (8.6)	2.5 (7.7)
	*95% CI*	(15.8, 22.8)	(−3.9, 26.9)	-	(−13.3, 0.6)	(−1.0, 6.8)	(−0.9, 5.9)
	*P*^f^	<0.001	0.098	-	0.059	0.140	0.140

^a^Nine hospitals and their linked community centres. MCHH: Maternal and Child Health Hospital, SMiH: St Michael’s Hospital, EGH: Ejura Government Hospital, APH: Agogo Presbyterian Hospital, SPH: St Patrick’s Hospital, KGH: Kogongo Government Hospital, SMaH: St Martin’s Hospital, KSH, Kumasi South Hospital, MGH: Mankranso Government Hospital.

^b^Three public universities and 2 health and nursing/midwifery training colleges. KNUST: Kwame Nkrumah University of Science and Technology, CHK: College of Health Kintampo, UHAS: University of Health and Allied Sciences, F-CHNTS: Community Health Nurses Training School – Fomena, CSUC: Christian Services University College.

^c^The eLearning course formed part of formal curriculum activities and timetabled in the MeLE study period.

^d^The eLearning course was introduced as an additional learning resource in the MeLE study period.

^e^Delivery models. MTC: Mobile Training Centre, OD: Online Delivery, ICW: Institutional Computer Workstation and MD: Mixed Delivery.

^f^Paired *t* test was performed.

^g^Includes one participant whose post-assessment score was much lower than pre-assessment score (−13.3), due to partially completing the post-assessment. After excluding this, overall: N = 13, Mean (SD) Pre = 35.8 (12.1), Mean (SD) Post = 35.5 (15.2), Diff (SD) = −0.3 (8.4), 95% CI = (−5.4, 4.8), *P* = 0.903; completed: N = 3, Mean (SD) Pre = 40.1 (9.9), Mean (SD) Post = 44.4 (11.9), Diff (SD) = 4.2 (2.1), 95% CI = (−0.9, 9.4), *P* = 0.072.

#### Knowledge gains between delivery models

When grouped knowledge gain by delivery models, the MTC group, KNUST and CHK in OD group obtained a significant knowledge gain from the course regardless of whether participants had completed the course, were in progress or did not complete it (Table 3). The participants in the MTC group, KNUST and CHK had scheduled self-directed training sessions with the course and, therefore, these participants’ learning time on the course was similar. Majority completed the course within the scheduled sessions. For those who did not complete the course, except two participants at KNUST who reported Internet issues, the rest reached nearly the end of the course. The differences between pre and post-assessments in the MTC group by completion status were: Completed 15.4%, In progress 14.9% and Not completed 19.3%. The differences in KNUST were: Completed 24.5%, In progress 26.6% and Not completed 11.5%; while that for CHK is Completed 14.8%. However, there was no significant increase in post-assessment score among the participants at UHAS in OD group.

For F-CHNTS in ICW and CSUC in MD groups, significant differences in knowledge gains were observed only for those who completed the course but not for those who were in progress or did not complete it. This differing response compared with the MTC group may be due to the latter in the MTC group and at KNUST and CHG in the OD group approaching learning more thoroughly and reaching nearly the end of the course while ICW and MD groups did not. In addition, the differences between the pre and post-assessments among those who completed the course at UHAS, F-CHNTS and CSUC were lower, ranging from −2.3% to 9.8%, than the MTC group or KNUST and CHK in OD group, which were from 14.8% to 24.5%.

#### Relevance to job/academic progression and knowledge gain

**Figure 2.**
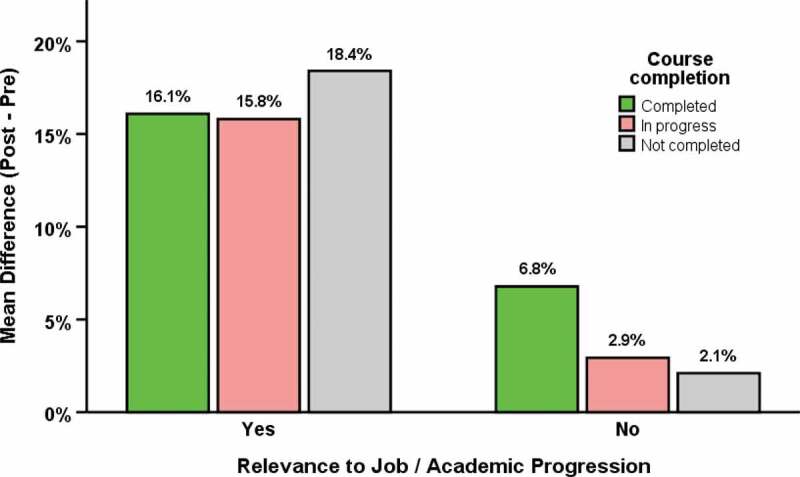
Differences in knowledge gains (post-pre assessments) by relevance to job/academic progression (Yes: relevant to job/academic progression – MTC group, KNUST and CHK in OD group; No: not relevant to academic progression – UHAS in OD, F-CHNTS in ICW and CSUC in MD groups). In the ‘relevant to job/academic progression’ group, (i) Completed: Mean difference = 16.1%, 95% CI (14.7, 17.5), *P* < 0.001; (ii) In progress: Mean difference = 15.8%, 95% CI (11.3, 20.3), *P* < 0.001; (iii) Not completed: Mean difference = 18.4%, 95% CI (15.0, 21.8), *P* < 0.001; (iv) Overall: Mean difference = 16.3%, 95% CI (15.1, 17.5), *P* < 0.001. In the ‘not relevant to job/academic progression’ group, (i) Completed: Mean difference = 6.8%, 95% CI (3.5, 10.1), *P* < 0.001; (ii) In progress: Mean difference = 2.9%, 95% CI (−0.04, 5.9), *P* = 0.053; (iii) Not completed: Mean difference = 2.1%, 95% CI (−0.3, 4.5), *P* = 0.086; (iv) Overall: Mean difference = 3.8%, 95% CI (2.2, 5.5), *P* < 0.001.)

Regardless of course completion status, the “relevant to job/academic progression” group performed significantly better in the post-assessment than pre-assessment by 16.3% (*95%* CI 15.1, 17.5; *P* < 0.001). In the ‘not relevant’ group, only those who completed the course did significantly better in the post-assessment but the difference is limited to 6.8% (*95%* CI 3.5, 10.1; *P* < 0.001). Relevance of the course to job/academic progression affected knowledge gain significantly irrespective of course completion (*P* < 0.001) (Figure 2 and Supplementary file 7).

### Delivery costs of MTC, OD, ICW and MD with malnutrition eLearning course vs. face-to-face training

Costs for training with the malnutrition eLearning course through the different delivery models are compared in Table 4, and with comparable face-to-face training. The OD and ICW models eliminated the costs for venue hire, trainee travel and refreshments. MTC incurred many of the costs associated to face-to-face training but saved on trainer cost and venue hire. The unit costs per participant for training with the malnutrition eLearning course through MTC, OD, ICW and MD were: £51.0, £2.2, £1.2 and £1.1, respectively. Although higher than other models, the unit cost for MTC is about half that of delivering face-to-face training in this setting, which amounts to £105.0 (1 GHS = 0.14 GBP).

**Table 4.** Cost comparison between face-to-face training delivery and different delivery models used for the malnutrition eLearning course^a.^

			Delivery model^b^				
	Unit cost (GHS)						
Items of money spent actually on	Unit	Cost	Face-to-face 3-day training^3^	MTC (9 institutions)	OD (3 institutions)	ICW (3 institutions)	MD (1 institution)
Number trained^4^	N/A		30	318	172	313	112
Staff cost^e^	Per diem	1000	9,000	40,500	1,800	1,800	600
Trainer travel cost^f^	Daily per person	100	300	8,100	900	900	300
Trainees travel cost^g^	Daily per person	20	1800	19,080	0	0	0
Refreshment^h^	Daily per person	100	9900	48,150	0	0	0
Venue hire^i^	Per training	1500	1500	0	0	0	0
**Total in GHS**			22,500	115,830	2,700	2,700	900
**Total in GBP**^j^			£3,150.0	£16,216.2	£378.0	£378.0	£126.0
**Unit cost for training per person (GHS/GBP)**			**750/£105.0**	**364.2/£51.0**	**15.7/£2.2**	**8.6/£1.2**	**8.0/£1.1**

^a^The costs for developing eLearning and face-to-face training have not been included.

^b^Delivery models. MTC: Mobile Training Centre, OD: Online Delivery, ICW: Institutional Computer Workstation and MD: Mixed Delivery.

^c^The cost was calculated on the assumption that the training will be delivered by external trainers.

^d^Number of participants trained, as the basis of unit cost.

^e^Per diem for OD and ICW (1 hour) = 200, MTC (0.5 day) = 500. Staff cost = unit cost x no of trainers/resource persons x number of days x number of institutions: face-to-face training: daily unit cost (1000) x 3 trainers x 3 days x 1 = 9000, MTC = half day unit cost (500) x 3 persons x 3 days x 9 institutions = 40,500, OD = 1 hour unit cost (200) x 3 persons x 1 day x 4 institutions = 1800, ICW = 1 hour unit cost (200) x 3 persons x 1 day x 3 institutions = 1800, MD = 1 hour unit cost (200) x 3 persons x 1 day x 1 institutions = 600.

^f^Trainer travel cost = unit travel cost x number of individuals x number of visits x number of institutions. Travel costs for: face-to-face training = 100 x 3 × 1 x 1 = 300, MTC = 100 x 3 × 3 x 9 = 8100. OD, ICW MD = 100 x 3 × 1 x 3 = 900 for OD, 900 ICW and 300 MD. The calculation of travel cost for MTC is made based on 3 days.

^g^Trainee travel cost = unit cost for travel x number of days x number of individuals. Trainee travel costs for: face-to-face training = 20 x 3 × 30 = 1800, MTC = 20 x 3 × 318 = 19,080.

Reimbursing trainees’ travel cost was relevant as participants came from different health centres to the district hospitals for the training.

^h^Refreshment cost = unit cost for refreshment x number of days x number of persons invited to training plus trainers. The refreshment cost for face-to-face raining = 100 x 3 × 33 = 9900, MTC = 50 x 3 × 321 (Unit cost for refreshment for MTC is different from face-to-face because training takes half a day).

^i^Venue cost may be 0 GHS if institutions have venue for training. Otherwise, cost will apply. For the MTC, a room/venue appropriate for self-directed learning (not suitable for face-to-face training) was made available by each participating healthcare institution.

^j^1 GHS = 0.14 GBP.

## Discussion

We have previously demonstrated that the malnutrition eLearning course improved knowledge, understanding and skills of health professionals in the diagnosis and management of children with SAM, leading to changes in clinical practice and improved confidence, and improved identification of SAM and almost all aspects of the WHO ‘Ten Steps’ of case-management, and reduced case-fatality [32,33]. This study further demonstrates that scaling up capacity/training in malnutrition management, a recognised challenge in most LMICs [22–26], is possible through eLearning if it is supported by contextually appropriate delivery and implemented as part of curriculum development or in-service training. Key strategies outlined by UNICEF to expand access to quality treatment of SAM include building national capacities and strengthening systems to support SAM management scale-up for which a well-trained workforce is essential [27]. Despite many calls for capacity building among health professionals in the management of malnutrition [28,34], taking the necessary steps to make this happen has not been easy. During MeLE in Ghana, the eLearning course was offered to 938 in-service and pre-service health professionals at 9 healthcare and 7 academic institutions. The numbers of institutions involved and participants trained in this study demonstrate the potential of the course as a scalable training solution for SAM management. The implementation process for the malnutrition eLearning course and findings from the evaluation also offer a practical guide for implementing available eLearning in general to increase access to training and to support its effective utilization in LMICs [1].

Online delivery is ideal for eLearning but this is only possible where Internet is accessible. In Ghana Internet limitations are common [35] and going online can be problematic [36]. Only 3 of the 16 institutions in MeLE had sufficient Internet capacity for online delivery. Limited computer and Internet access however can be overcome. The 9 healthcare institutions in the study had neither workstations nor Internet access. Through setting up a temporary training facility (MTC) using the offline malnutrition eLearning course on the research team’s and participants’ own computers, the participants were able to take the course. And so were the participants at 4 academic institutions through ICW and MD. The results showed that participants’ completion of the course was not determined by delivery models nor access to computer (*P* = 0.398) and/or Internet (*P* = 0.134) at home. This indicates that the four delivery models implemented in this study were different but offered equivalent learning/training opportunities to the participants. Hospitals/health centres in Ghana organize periodic in-service training, and nationally there is a drive for academic institutions to have workstations equipped with computers [37]. Therefore, this result is important as it offers a way forward for the management of malnutrition and other areas requiring scalable training solutions in Ghana and other LMICs.

Previous studies suggested that recognition of eLearning, integrated into curricula and/or recognized as CPD, influences its completion and benefits gained from it [9,10], and this was confirmed by the study. The benefits derived from the malnutrition eLearning course were determined by relevance of the course to job and how it was integrated into academic curricula. Two academic institutions in the OD group, CHK and KNUST where the course was integrated as required curriculum activities in the evaluation period, had course completion rates of 100% and 79.4%, respectively. However, only 26.7% of UHAS participants in the OD group where the course was offered as additional learning completed the course.

Course completion was much higher among participants in ‘Relevant to job/academic progression’ group (*P* < 0.001), irrespective of computer access (yes 80.1%, no 81.3%) or internet access (yes 76.2%, no 83.4%) at home than those in ‘Not relevant’ (computer access: yes 33.3%, no 24.1%; Internet access: yes 29.7%, no 29.5%). Knowledge gained from the course was also affected by relevance of the course to participant jobs or academic progression. The gained knowledge in the ‘Relevant to job/academic progression’ group was 12.5% higher than the ‘Not relevant’ group (*95%* CI 10.2, 14.7; *P* < 0.001).

Two academic institutions, KNMTC and KNTC, implemented the course as extra, optional, learning. Both took part in the pre-assessment but, according to staff, the students complained of having no readily accessible internet access. We surmise that timetabled activities were prioritised for workstations and, as it was optional learning, it was difficult for the students to access computer and Internet during study hours and they also had less motivation to complete the course. The study team subsequently organised an offline version for the institutions but it was then too late for the students to complete the training in the study period. We excluded these institutions from the analysis as no post-assessment scores were available. These observations perhaps underscore the importance of timetabling learning sessions with any eLearning to ensure participants have time to access and take part, as well as the need to strengthen capacity for independent learning, if eLearning is to be effective.

Major obstacles to in-service training of healthcare workforce are the delivery cost of centralized tutor-based training, shortage of experienced trainers, inadequate supply of training materials, poor follow-up and supportive supervision, the difficulty of releasing essential staff for off-site training and costs of per diem travel and accommodation [38,39]. The cost analysis undertaken suggests that implementing the malnutrition eLearning can reduce the training cost on SAM management as well as increase access to training opportunities. OD, ICW, MD are most cost-effective, needing a minimal staff time for eLearning introduction. MTC incurs a higher cost requiring scheduled sessions even if the training is self-directed. However, it still is less than half the cost of delivering face-to-face onsite training. For eLearning that is proven to be effective, these different models should be considered, especially in LMICs, to scale up training of healthcare workforce. The eLearning course was developed in the UK and the initial cost for developing the course was high (£40,000 for a 6–9 hour highly interactive, media-rich eLearning), and development costs may limit the potential of eLearning. However, the main rationale for this paper is to show the feasibility of utilizing available eLearning for scalable capacity building. Furthermore, with many eLearning courses, including massive open online courses (MOOCs), freely available for institutions and individuals to take advantage of, new eLearning development should be considered only if there is no available eLearning. If new eLearning is to be developed, then the initial development costs will have to be addressed. Costs for computers were not included as our aim was to utilize existing resources, which proved possible. Hiring or purchasing computers would increase implementation costs.

This study has limitations. Course completion was self-reported by participants, and some may have misreported. Online course completion was tracked in a database but not for the offline version. Participation in MeLE was voluntary and kept confidential; therefore, we consider participants reported their completion status correctly. The post-assessment score for those who completed the course at UHAS was lower than their pre-assessment score. UHAS post-study participation rate was low (15/65, 23.1%) and only 4 completed the course. We do not know the reasons for their low study participation but it is likely that the low participation rate, low course completion and no significant improvement between pre and post-assessments are associated.

## Conclusion

The malnutrition eLearning course makes global capacity building in malnutrition management achievable and effective. Our experience in implementing the course in Ghana suggests that available eLearning can be utilized successfully to scale up in-service and pre-service training in LMICs if contextually appropriate delivery is considered and implemented to suit institutional IT readiness and is introduced as a core part of curricula or is implemented within in-service training for those with relevant job responsibilities.

## Supplementary Material

Supplemental Material

Supplemental Material

Supplemental Material

Supplemental Material

Supplemental Material

Supplemental Material

Supplemental Material

## Data Availability

The dataset from which the analyses have been made will be made available upon request.

## Paper context

Building capacity for malnutrition management is a recognised global challenge. eLearning can offer scalable training at low cost. In LMICs this requires effective implementation to overcome infrastructure challenges and ensure utilization. This study reports training of health professionals at 16 healthcare and academic institutions in Ghana using the malnutrition eLearning course through implementing four different delivery models. Course completion rates and benefits realized were significant, demonstrating effective eLearning implementation for capacity building at scale.
