# Supplementary material for: Implementing effective eLearning for scaling up global capacity building: findings from the malnutrition elearning course evaluation in Ghana
Source: Glob Health Action. 2020 Oct 22;13(1):1831794. doi: 10.1080/16549716.2020.1831794 (PMC7595220; doi:10.1080/16549716.2020.1831794)
Supplement: Supplemental Material [file ZGHA_A_1831794_SM8649.docx]

**APPENDIX**

**Supplementary file 1. Steps taken to design and implement the malnutrition eLearning course delivery**

**Step 1**

Identifying factors that would influence the course delivery in the study contexts

**Step 3**

Devising and mapping suitable delivery models for the course

**Step 4**

Implementing the different delivery models

**Step 2**

Determining suitable solutions to address the factors identified in Step 1
