# Supplementary figures and images for: Implementing effective eLearning for scaling up global capacity building: findings from the malnutrition elearning course evaluation in Ghana
Source: Glob Health Action. 2020 Oct 22;13(1):1831794. doi: 10.1080/16549716.2020.1831794 (PMC7595220; doi:10.1080/16549716.2020.1831794)

**Supplementary file 2. Process of mapping the 4 delivery models (OD, ICW, MTC and MD)**


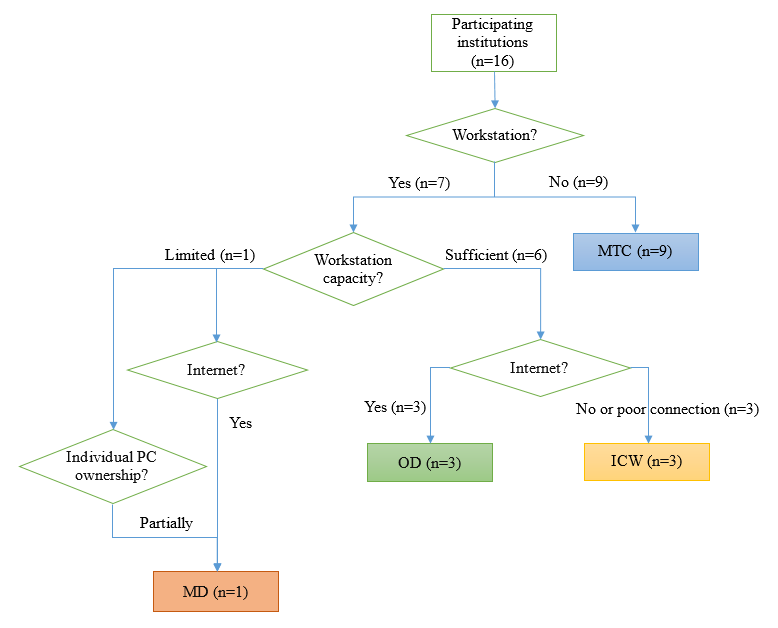

Supplement: Supplemental Material [file ZGHA_A_1831794_SM8647.docx]
