# Supplementary material for: Implementing effective eLearning for scaling up global capacity building: findings from the malnutrition elearning course evaluation in Ghana
Source: Glob Health Action. 2020 Oct 22;13(1):1831794. doi: 10.1080/16549716.2020.1831794 (PMC7595220; doi:10.1080/16549716.2020.1831794)
Supplement: Supplemental Material [file ZGHA_A_1831794_SM8628.pdf]

Supplementary file 3. Paired pre- and post-assessment tools

Pre-assessment questions	Difficulty	No	Post-assessment questions	Difficulty
**Module 1**			**Module 1**	
What is severe acute malnutrition? Select the best definition.<br><br>1) It is being "badly nourished" and is used to describe undernutrition.<br>2) It is severe wasting and/or stunting.<br>3) It is growth faltering or over-weight.<br>4) It is severe wasting and/or oedema.		1	What is the definition of severe acute malnutrition?<br><br>1) Being "badly nourished".<br>2) Having severe wasting and/or stunting.<br>3) Having a low weight for age<br>4) Having severe wasting and/or oedema.	
Which of these are common causes of death in children with severe acute malnutrition? Tick all that apply.<br><br>1) Hypoglycaemia<br>2) Hyperthermia<br>3) Missed infection<br>4) Iron deficiency		2	Children with severe acute malnutrition are at high risk of dying from which of these conditions? Tick all those that apply.<br><br>1) Hyperglycaemia<br>2) Hypothermia<br>3) Overhydration<br>4) Heart failure<br>5) Missed infection	
Why do severely malnourished children need different care from well-nourished children? Tick all that apply.<br><br>1) Because of hidden changes in the body.<br>2) Because they are stunted.<br>3) Because infections may be hidden.<br>4) Because heart and kidney function is impaired.		3	When a child's energy and nutrient intakes are insufficient, physiological and metabolic changes take place in the child's body to conserve energy. What is this process called?	
Which statements are correct?<br><br>1) Acute malnutrition is common in famine.<br>2) Malnutrition is linked to poor feeding practices, poor hygiene and illness.<br>3) Inadequate nutrition during pregnancy can lead to poor appetite in the offspring.<br>4) Chronic malnutrition leads to stunting.		4	Which statements are correct?<br><br>1) Chronic malnutrition is the result of a prolonged period of inadequate nutrition.<br>2) Acute malnutrition leads to stunting.<br>3) Chronic malnutrition leads to wasting.<br>4) Underweight or low weight-for-age can result from acute or chronic malnutrition.	
What visible changes may take place when children become severely malnourished?		5	Choose all visible changes that may arise as a result of severe acute malnutrition.	

1) Swollen feet<br>2) Flaky paint dermatosis<br>3) Hair becomes thin and discoloured<br>4) Body tends to conserve energy<br>5) Miserable and apathetic			1) Poor appetite<br>2) Dehydration<br>3) Loose skin<br>4) Irritable<br>5) Stops growing	
Which of the following are invisible changes that take place in children with severe acute malnutrition?<br><br>1) Liver makes glucose less easily<br>2) Heart pumps slower<br>3) Loss of appetite<br>4) Increased activity inside cells to support vital organs<br>5) Oedema		6	Choose the invisible changes that take place in severely malnourished children.<br><br>1) Poor appetite<br>2) Potassium leaks out of cells<br>3) Building up of muscle and tissues to fight infections<br>4) Decreased response to infections<br>5) Increased work for some organs	
Which of the following conditions, if present, would denote severe malnutrition 'with complications'?<br><br>1) High fever<br>2) Colic<br>3) Respiratory distress<br>4) Poor appetite<br>5) Profuse diarrhoea		7	Which of the following conditions denote severe acute malnutrition 'with complications'?<br><br>1) Severe anaemia<br>2) Bulimia<br>3) Poor appetite<br>4) Generalised oedema<br>5) Loose stools	
Moderate acute malnutrition can be assessed by which of these? Tick all that apply.<br><br>1) Mid Upper Arm Circumference (MUAC) <115 mm for children aged 6 - 59 months<br>2) Weight-for-height between -2 and -3 SD<br>3) Weight-for-height between - 1 and -2 SD<br>4) MUAC between 115 and 125 mm for children aged 6 - 59 months		8	Severe acute malnutrition can be assessed by which of these? Tick all that apply.<br><br>1) Mid Upper Arm Circumference (MUAC) <115 mm for children aged 6 - 59 months<br>2) Presence of bilateral oedema<br>3) Weight-for-height <-2 SD<br>4) MUAC between 115 and 125 mm for children aged 6 - 59 months	

Sheema is a 24 month old girl. Her length and weight are 67 cm and 3.9 kg. Which category does she meet? Click here for the WHO weight-for-height girl chart.<br><br>1) Not malnourished<br>2) Mild acute malnutrition<br>3) Moderate acute malnutrition<br>4) Severe acute malnutrition<br><br>*Note: Provide the printed WHO-weight-for-height girl chart if using the paper-based version.*		9	Kofi is a 54 month old boy. His height and weight are 110 cm and 18.5 kg. Which category does he meet? Click here for the WHO weight-for-height boy chart.<br><br>1) Not malnourished<br>2) Mild acute malnutrition<br>3) Moderate acute malnutrition<br>4) Severe acute malnutrition<br><br>*Note: Provide the printed WHO-weight-for-height boy chart if using the paper-based version.*	
How should children with severe acute malnutrition (SAM) be treated? Select all correct answers.<br><br>1) They should be rehydrated quickly to prevent possible heart failure.<br>2) They should be fed differently from well-nourished children because their gut and liver cannot cope with normal meals.<br>3) Specific nutrients should be given to correct micronutrient deficiencies.<br>4) They need to be kept warm all the times.<br>5) They should be given iron in the stabilisation phase of treatment.		10	How should children with SAM be treated? Select all correct answers.<br><br>1) They should be rehydrated more slowly than for well-nourished children to prevent fluid overload and possible heart failure.<br>2) They should be fed normal foods but more frequently to help their organs recover.<br>3) Specific nutrients should be given to correct electrolyte imbalances.<br>4) Once they become stabilised, they should be kept warm.<br>5) They should be given iron in the rehabilitation phase of treatment.	
**Module 2 pre-assessment questions**			**Module 2 post-assessment questions**	
Which of these indices are best for screening for acute malnutrition in communities?<br><br>1) Mid Upper Arm Circumference (MUAC)<br>2) Weight-for-height<br>3) Oedema<br>4) Length-for-age<br>5) Weight-for-age		11	Which of these indices can be used to assess the severity of acute malnutrition?<br><br>1) Mid Upper Arm Circumference (MUAC)<br>2) Weight-for-height<br>3) Oedema<br>4) Length-for-age<br>5) Weight-for-age	

Which of the following statements is true?<br><br>1) Length-for-age is used for children below 2 years of age and is measured with the child lying down.<br>2) Height-for-age is used for children over 2 years of age and is measured with the child standing.<br>3) Length is the same as height but just taken in a different position.<br>4) For a child aged 2 years or older who is too weak to stand, his or her length should be measured and 0.7 cm be added.		12	Which of the following statements is true?<br><br>1) Length-for-age is used for children below 2 years of age and is measured lying or standing.<br>2) Length and height give the same reading.<br>3) For a child aged 2 years or older who is too weak to stand, his or her length should be measured and 0.7 cm be added.<br>4) If a child aged 2 years or older is too weak to stand, his or her length should be measured instead of height and 0.7 cm subtracted.	
Hassan is an 18 month year old boy. He is 65 cm in length and weighs 4.8 kg. Which of these indicators should you use to determine if he has severe acute malnutrition?<br><br>1) Bilateral pitting oedema<br>2) Visible severe wasting<br>3) Length-for-age <-3 SD<br>4) Weight-for-age < -3 SD<br>5) Weight-for-length <-3 SD		13	Ola is a 13 month old girl. She weighs 7.7 kg. Her MUAC is 110 mm. Which indicators or clinical signs would you use to determine if she has severe acute malnutrition?<br><br>1) Bilateral pitting oedema<br>2) MUAC <115 mm<br>3) Weight-for-age <-3 SD<br>4) Weight-for-length <-2 SD	
Which indicator should you use to identify children who are stunted due to prolonged under-nutrition or repeated illness?<br><br>1) Height/length-for-age<br>2) Weight-for-age<br>3) Weight-for-length<br>4) MUAC		14	Which statement is false?<br><br>1) Height/length-for-age is used to identify children who are stunted.<br>2) A low height-for-age is due to prolonged under-nutrition or repeated illness.<br>3) A low height-for-age is used to identify children with acute malnutrition<br>4) Height/length-for-age is the length or height in comparison to that of an average healthy child of the same age.	

When is it most appropriate to use the height/length-for-age index?<br><br>1) In nutritional surveys to determine the prevalence of acute malnutrition<br>2) For growth monitoring<br>3) In clinics to assess severity of malnutrition<br>4) In nutritional surveys to determine levels of stunting<br>5) To assess wasting		15	When is it most appropriate to use the weight-for-age index?<br><br>1) In surveys of nutritional status<br>2) For growth monitoring<br>3) In clinics to assess severity of malnutrition<br>4) In nutritional surveys to determine levels of stunting<br>5) To assess wasting	
Which of the following statements are true?<br><br>1) Grade +++ (severe) is generalised oedema including feet, legs, hands, arms and face).<br>2) Oedema is the retention of fluid in tissues of the body.<br>3) Oedema can be examined by holding a child's hands with your thumbs on top, press the top of the child's hands gently for 10 seconds and lift your thumbs.<br>4) It may be mistaken for fatness.<br>5) If a child has severe oedema but is not wasted, the child can be treated in the community.		16	Which of the following statements are true?<br><br>1) Oedema is partly the result of malfunctioning of the sodium-potassium pump in cells.<br>2) Children with oedema are at low risk of mortality.<br>3) Grade +++ (severe) is oedema of the feet plus lower legs, hands and lower arms.<br>4) Oedema is a clinical sign of severe acute malnutrition.<br>5) Children with severe oedema need to be treated in hospital or in a stabilisation centre.	
Which of the following statements is false?<br>1) Dehydration in SAM children requires special consideration because the usual signs (slow skin pinch, dry mouth and no tears) may be present even in the absence of dehydration.<br>2) Rehydration should continue until the skin pinch becomes normal.<br>3) Dehydration in SAM children needs to be treated with Rehydration Solution for Malnutrition (ReSoMal).<br>4) Watery stools may continue after dehydration has been corrected.		17	Which of the following statements is false?<br>1) Intravenous fluids are helpful in stopping watery stools.<br>2) It is difficult to tell if a SAM child is responding to rehydration fluid, as the signs of dehydration may remain even when the child is fully rehydrated.<br>3) Stop rehydration if a SAM child shows signs of fluid overload.<br>4) Signs of hydration are passing urine, faster skin pinch, tears, moist mouth and less thirsty.	

Where should a SAM child with poor appetite be treated?<br><br>1) Inpatient facility<br>2) Outpatient clinic<br>3) Community rehabilitation centre<br>4) At home		18	Why must a SAM child with a poor appetite be admitted into an inpatient facility? Tick all that apply.<br><br>1) Because feeding is difficult to administer at home and the child may not eat enough.<br>2) Because lack of appetite is a sign of profound metabolic changes or an infection.<br>3) Because the child needs a special, energy-dense food.<br>4) Because it often leads to heart failure.	
Which of the following statements is false regarding height measurement?<br><br>1) Two measurers are needed for accurate results - one to position the child and the other to write down the measurement.<br>2) Always remove hair ornaments and undo braids.<br>3) Check the child's heels, buttocks, shoulder blades and back of the head touch the vertical board.<br>4) If a child is above 2 years but cannot stand, measure length and subtract 0.7 cm.<br>5) Make sure the equipment is correctly calibrated.		19	Which of the following statements is false regarding weight measurement?<br><br>1) If using pan scales, place a clean, dry cloth in the pan and set to zero before weighing the baby.<br>2) If using a scale with pants, zero the scale before attaching the pants.<br>3) Remove the child's clothes before weighing him/her.<br>4) When the weight is stable, read to nearest 10g.<br>5) Check if the scale has returned to zero after taking a child's weight.	
**Module 3 pre-assessment questions**			**Module 3 post-assessment questions**	
Which of the following criteria would you use to decide if a SAM child should receive inpatient or outpatient treatment?<br><br>a. Severity of wasting from weight-for-height or MUAC<br>b. Signs of complications<br>c. Weight-for-age<br>d. Grade of oedema<br>e. Appetite<br>f. Presence of oedema<br>g. Age<br><br>1) a, b, d, e and g<br>2) a, b, e and f<br>3) b, c, d and e<br>4) a, b, f and g		20	Who can be managed in a community based programme?<br><br>1) A 3 month old baby whose MUAC is 120 mm with oedema grade + and no loss of appetite.<br>2) A 13 month old girl whose weight-for-length is <-3SD with oedema grade +++. She has no appetite and is apathetic.<br>3) A boy whose age is unknown. His MUAC is 114 cm and he has no oedema and no loss of appetite.	

Which require inpatient care?<br><br>1) Generalised oedema<br>2) Weight-for-age <-3SD<br>3) Weight-for-height <-2SD with appetite loss<br>4) Visible severe wasting and not alert<br>5) Bilateral pitting oedema in feet and legs		21	Which can be managed in community-based care?<br><br>1) Visible severe wasting and clinically unwell<br>2) Generalised oedema<br>3) Bilateral pitting oedema in feet and legs<br>4) Weight-for-age <-3SD<br>5) SAM with difficulty in breathing	
Seth is an 18 month old boy. He is 65 cm in length and weighs 4.8 kg. His length-for-age is < -3 SD and he has loss of appetite. Where should he be treated?<br><br>1) Inpatient facility<br>2) Outpatient facility<br>3) Community based management facility<br>4) At home		22	Rob is a 5 month old boy. His grandmother brought him to hospital after an episode of diarrhoea. He is diagnosed with SAM but does not have oedema. Where should he be treated?<br><br>1) Inpatient facility<br>2) Outpatient facility<br>3) Community based management facility<br>4) At home	
Below are the WHO 10 steps rearranged. Which steps fall into the stabilisation phase?<br><br>1) Prepare for discharge and follow up<br>2) Treat and prevent dehydration<br>3) Treat and prevent hypothermia<br>4) Treat and prevent hypoglycaemia<br>5) Treat infections<br>6) Give catch up diet for rapid growth<br>7) Correct electrolyte imbalance<br>8) Start cautious feeding<br>9) Provide loving care and cognitive stimulation<br>10) Correct micronutrient deficiencies		23	Below are the WHO 10 steps rearranged. Which steps fall in the rehabilitation phase?<br><br>1) Treat infections<br>2) Correct micronutrient deficiencies.<br>3) Treat and prevent hypothermia<br>4) Give catch up diet for rapid growth<br>5) Prepare for discharge and follow up<br>6) Start cautious feeding<br>7) Treat and prevent hypoglycaemia<br>8) Correct electrolyte imbalance<br>9) Provide loving care and cognitive stimulation<br>10) Treat and prevent dehydration	
Which of the WHO 10 steps can be managed in the community?<br><br>1) Treat and prevent hypothermia<br>2) Correct electrolyte imbalance<br>3) Correct micronutrient deficiencies<br>4) Start cautious feeding<br>5) Treat and prevent dehydration		24	Which of the WHO 10 steps must be managed in an inpatient facility?<br><br>1) Treat and prevent hypothermia<br>2) Correct electrolyte imbalance<br>3) Correct micronutrient deficiencies<br>4) Start cautious feeding<br>5) Start sensory stimulation	

What are the principles of community-based management of acute malnutrition?<br><br>1) Timely management<br>2) Maximum access and coverage of treatment<br>3) Appropriate medical care and nutritional rehabilitation<br>4) Care as long as needed<br>5) Critical care		25	Which of these are essential components of community-based management of acute malnutrition?<br><br>1) Community outreach<br>2) Supplementary feeding programme<br>3) Outpatient care for SAM children with no complications<br>4) Inpatient care or stabilisation centres<br>5) Medical equipment to treat dehydration and hypoglycaemia	
A child called Adam was brought to a hospital. His age was unknown. He is 74.0 cm 6.0 kg and weight-for-length <-4SD. He is drowsy and lethargic and cannot be roused. What condition might Adam have?<br><br>1) Heart failure<br>2) Hypoglycaemia<br>3) Hypernatremia<br>4) Dehydration<br>5) Electrolyte imbalance		26	What is hypoglycaemia?<br><br>1) Low level of glucose in the blood<br>2) Low level of potassium in the blood<br>3) High level of sodium in the blood<br>4) High level of glucose in the blood<br>5) High level of potassium in the blood	
Adam was brought to a hospital. His age was unknown. He is 74.0 cm 6.0 kg and weight-for-length <-4SD. He is lethargic and cannot be roused. How should Adam be treated?<br><br>1) Give 30 ml of 10% sterile glucose solution immediately IV.<br>2) Give 60 ml of ReSolMal immediately IV.<br>3) Give 50 ml of 10% glucose solution immediately IV or by mouth.<br>4) Dissolve 5g of sugar in 50 ml of water and give it to Adam.		27	Below are treatment options for hypoglycaemia if a child is conscious. Which are correct?<br><br>1) 50 ml of 10% glucose solution by mouth<br>2) 50 ml of F100<br>3) 50ml ReSoMal<br>4) 10% glucose solution 5 ml/kg body weight IV<br>5) F75 immediately and every 30 minutes	

Which of the following statements about feeding severely malnourished children are true?<br><br>1) Feed SAM children small amounts frequently.<br>2) Feed A SAM child with generalised oedema 130 ml starter formula/kg/day.<br>3) Feeding should start immediately because SAM children are at risk of hypothermia.<br>4) SAM children who are breastfed should receive the prescribed amount of F75 and encouraged to breastfeed.<br>5) In the stabilisation phase, SAM children should be fed every 4 hours (6 feeds per day).		28	In the stabilisation phase how should feeding a SAM children to be managed? Select correct statements.<br><br>1) The feeding should provide 100kcal/kg/day and 1-1.5g protein/kg/day.<br>2) Give F100 for the first few days every 2 - 3 hours.<br>3) Give SAM children with no oedema 130 ml F75/kg/day to meet the energy and protein targets.<br>4) For SAM children with generalised oedema, give 130ml F75 /kg/day.<br>5) If SAM children are breastfed, breastfeeding should stop while they receive the prescribed amount of F75 to maintain their daily energy and protein intake.	
Sheema was admitted into an inpatient facility for SAM with complications. She is 2 years, 67.0 cm, 4.0 kg and weight-for-length <-4SD.<br><br>Calculate how much F75 Sheema should be given a) in a day and b) per feed, if fed 3-hourly.<br><br>_______ ml per day, _________ ml per feed		29	Fatima is 1 year 8 months old, 67.0 cm and 6.5 kg. Her weight-for-length is between -1 and -2SD. She has generalised oedema.<br><br>Calculate how much F75 Fatima should be given a) in a day and b) per feed, if fed 3-hourly (to nearest whole number).<br>_______ ml per day, _________ ml per feed	
Which of the following electrolytes are in deficit in children with severe acute malnutrition?<br><br>1) Potassium<br>2) Magnesium<br>3) Chloride<br>4) Sodium		30	Which of the following statements about electrolyte imbalance in SAM children is true?<br><br>1) SAM children have too little sodium and too much potassium in their bodies.<br>2) Signs of electrolyte imbalance are oedema, apathy, and poor appetite.<br>3) Diuretics are good for treating oedema and help correct electrolyte imbalance.<br>4) Rehydrate with ReSoMal and prepare food with salt.	
Which of the following statements about treatment of SAM children is false?		31	Which of the following statements about treatment of SAM children is false?	

1) All SAM children should be given antibiotics even if they do not show signs of infection.<br>2) Hypothermia is measured as rectal temperature below 35.5^{0}C.<br>3) The aim of the rehabilitation phase is to stabilise damaged organs.<br>4) If a SAM child has severe anaemia, whole blood 10 ml/kg body weight should be given slowly over 3 hours.<br>5) Micronutrients that are likely to be deficient in SAM children are vitamin A, zinc, copper and folic acid.			1) To treat severe anaemia, give furosemide 1 ml/kg IV at the start of the transfusion.<br>2) The aim of the rehabilitation phase is to rebuild wasted tissue.<br>3) When rehydrating a SAM child, monitor pulse and respiratory rates to guard against overhydration.<br>4) Give a broad-spectrum antibiotic even if there is no sign of infection.<br>5) Give multivitamin supplement, folic acid 1 mg/d, Zinc 2 mg/kg/d, copper 0.3 mg/kg/d and iron 3 mg/kg/d for at least the first 2 weeks of treatment.	
Which of the following statements are false?<br><br>1) Ready-to-Use Therapeutic Food (RUTF) can be used instead of F100 in SAM management.<br>2) F100 is more energy dense thanF75 but has a similar protein content.<br>3) F100 is a milk-based formula containing 100 kcal and 2.9 g protein/100 ml.<br>4) F100 is used in both SAM management and supplementary feeding programmes.<br>5) A child with severe acute malnutrition is ready to transfer to the rehabilitation phase when appetite returns and most of the oedema has gone.		32	Which of the following statements is true?<br><br>1) If a SAM child has dehydration, F100 should be used instead of F75 in the stabilisation phase.<br>2) Ready-to-Use Therapeutic Food (RUTF) is difficult to store, so its use is limited to health facilities.<br>3) In the stabilisation phase, the target weight gain is > 10 g gain/kg/d and the target intakes are 150-220kcal/kg/d and 4-6 protein/kg/d<br>4) After 3 transition days, give as much F100 or RUTF as a SAM child can eat at least 7 times in 24 hours.<br>5) Because F100 can be easily prepared at home, it is used for supplementary feeding programmes.	
