# Supplementary material for: Implementing effective eLearning for scaling up global capacity building: findings from the malnutrition elearning course evaluation in Ghana
Source: Glob Health Action. 2020 Oct 22;13(1):1831794. doi: 10.1080/16549716.2020.1831794 (PMC7595220; doi:10.1080/16549716.2020.1831794)
Supplement: Supplemental Material [file ZGHA_A_1831794_SM8616.docx]

## Supplementary file 4. Malnutrition eLearning development cost in context

## eLearning development costing

The development cost for eLearning is determined by many factors and these include requirements (user, functions, data, design – who it is for, what it is for, what it should do, what data it should capture, how it should appear and work), constraints (technologies, platform and IT available to the provider and target users), delivery (what devices, browsers, etc. should be supported) and contextual environments (where and how the eLearning will be used). The cost is also affected by whether there is an in-house eLearning team who forms part of the eLearning development project or not.

Because of these, eLearning development is often approached in two different ways. The first is to design and develop new eLearning based on the requirements captured and design specified, and the other is to design eLearning within the available funding.

The items typically considered for costing are: creative learning solution, instructional/learning design, technologies used (low or high fidelity such as simulation and immersive environment), level of interactivity, number and complexity of media, platform requirements, learning time. The cost for project/team management is also considered.

**Malnutrition eLearning development cost in context**

For the malnutrition eLearning course development, we believe it is important to consider the cost in its development context. We reported to have spent £40K for 6 – 8 hours of highly interactive eLearning. The University of Southampton Faculty of Medicine had an eLearning development and research team, specialized in developing customized, innovative learning solutions in medicine and health science. Prior to the malnutrition eLearning course development, the team had designed and developed a bank of reusable programming codes [1] so that the team could design and implement customized eLearning solutions quickly and at low cost. Next, the team had developed a customized nutrition eLearning portal and nutrition courses (funded by the Department of Health) [2,3]. The overarching design solution devised for the nutrition courses was applied to the malnutrition eLearning course. The course implementation was supported by the bank of reusable programming codes which minimized the development of new coding required and shortened the implementation time and associated cost, and the course was offered through the nutrition eLearning portal hosted in a University server. These minimized the course development and operational costs, allowing the team to spend the available budget on interactive activities, media creation and customization of coding to optimize user experience in LMIC where bandwidth was low.

## Hammerton M, Choi S, Ke G, et al. Improving Efficiency of Bespoke eLearning Resource Development with Reusable ActionScript 3 Classes. In J. Herrington & C. Montgomerie (Eds.), Proceedings of ED-MEDIA 2010--World Conference on Educational Multimedia, Hypermedia & Telecommunications *(*pp. 3040-3045). Toronto, Canada ISBN 978-1-880094-81-5.

1. Ke S, Choi S, Ault E. Technical Solutions for Competency-Based eLearning for Public Health Nutrition. In J. Herrington & C. Montgomerie (Eds.), Proceedings of ED-MEDIA 2010--World Conference on Educational Multimedia, Hypermedia & Telecommunications (pp. 3886-3895). Toronto, Canada ISBN 978-1-880094-81-5
2. Choi S, Nestel P, Ke G, et al. Adaptive Nutrition eLearning Solution for Existing and Future Health Care Professionals. In T. Bastiaens, J. Dron & C. Xin (Eds.), Proceedings of E-Learn 2009--World Conference on E-Learning in Corporate, Government, Healthcare, and Higher Education (p. 194). Vancouver, Canada ISBN 978-1-880094-76-1
