# Supplementary material for: Implementing effective eLearning for scaling up global capacity building: findings from the malnutrition elearning course evaluation in Ghana
Source: Glob Health Action. 2020 Oct 22;13(1):1831794. doi: 10.1080/16549716.2020.1831794 (PMC7595220; doi:10.1080/16549716.2020.1831794)
Supplement: Supplemental Material [file ZGHA_A_1831794_SM8614.docx]

**Supplementary file 5. Course completion and access to computer and Internet at home**

|  | | **Access to computer** | | | **Access to Internet** | |
| --- | --- | --- | --- | --- | --- | --- |
|  |  | **Yes** | | **No** | **Yes** | **No** |
| **Overall** | *Completed* | 197 (66.8%) | | 131 (64.9%) | 118 (60.8%) | 209 (69.4%) |
|  | *In progress* | 58 (19.7%) | | 35 (17.3%) | 41 (21.1%) | 52 (17.3%) |
|  | *Not completed* | 40 (13.6%) | | 36 (17.8%) | 35 (18.0%) | 40 (13.3%) |
|  | ***Total*** | **295 (100%)** | | **202 (100%)** | **194 (100%)** | **301 (100%)** |
|  | ***P***^1^ | **0.398** | | | **0.134** | |
| **Relevant to job/academic progression** | *Completed* | 169 (80.1%) | 117 (81.3%) | | 99 (76.2%) | 186 (83.4%) |
|  | *In progress* | 24 (11.4%) | 14 (9.7%) | | 17 (13.1%) | 21 (9.4%) |
|  | *Not completed* | 18 (8.5%) | 13 (9.0%) | | 14 (10.8%) | 16 (7.2%) |
|  | ***Total*** | **211 (100%)** | **144 (100%)** | | **130 (100%)** | **223 (100%)** |
|  | ***P***^1^ | **0.880** | | | **0.246** | |
| **Not relevant to job/academic progression** | *Completed* | 28 (33.3%) | 14 (24.1%) | | 19 (29.7%) | 23 (29.5%) |
|  | *In progress* | 34 (40.5%) | 21 (36.2%) | | 24 (37.5%) | 31 (39.7%) |
|  | *Not completed* | 22 (26.2%) | 23 (39.7%) | | 21 (32.8%) | 24 (30.8%) |
|  | ***Total*** | **84 (100%)** | **58 (100%)** | | **64 (100%)** | **78 (100%)** |
|  | ***P***^1^ | **0.212** | | | **0.955** | |

^1^ Chi-square test was performed.
