# Supplementary material for: Implementing effective eLearning for scaling up global capacity building: findings from the malnutrition elearning course evaluation in Ghana
Source: Glob Health Action. 2020 Oct 22;13(1):1831794. doi: 10.1080/16549716.2020.1831794 (PMC7595220; doi:10.1080/16549716.2020.1831794)
Supplement: Supplemental Material [file ZGHA_A_1831794_SM8613.docx]

**Supplementary file 6. Course completion and relevance to job/academic progression**

|  | | **Relevance to job/academic progression** | |
| --- | --- | --- | --- |
|  |  | **Yes** | **No** |
| **Access to computer** | *Completed* | 169 (80.1%) | 28 (33.3%) |
|  | *In progress* | 24 (11.4%) | 34 (40.5%) |
|  | *Not completed* | 18 (8.5%) | 22 (26.2%) |
|  | ***Total*** | **211 (100%)** | **84 (100%)** |
|  | ***P***^1^ | **<0.001** | |
| **No access to computer** | *Completed* | 117 (81.3%) | 14 (24.1%) |
|  | *In progress* | 14 (9.7%) | 21 (36.2%) |
|  | *Not completed* | 13 (9.0%) | 23 (39.7%) |
|  | ***Total*** | **144 (100%)** | **58 (100%)** |
|  | ***P***^1^ | **<0.001** | |
| **Overall** | *Completed* | 286 (80.6%) | 42 (29.6%) |
|  | *In progress* | 38 (10.7%) | 55 (38.7%) |
|  | *Not completed* | 31 (8.7%) | 45 (31.7%) |
|  | ***Total*** | **355 (100%)** | **142 (100%)** |
|  | ***P***^1^ | **<0.001** | |
|  | | **Relevance to job/academic progression** | |
|  |  | **Yes** | **No** |
| **Access to Internet** | *Completed* | 99 (76.2%) | 19 (29.7%) |
|  | *In progress* | 17 (13.1%) | 24 (37.5%) |
|  | *Not completed* | 14 (10.8%) | 21 (32.8%) |
|  | ***Total*** | **130 (100%)** | **64 (100%)** |
|  | ***P***^1^ | **<0.001** | |
| **No access to Internet** | *Completed* | 186 (83.4%) | 23 (29.5%) |
|  | *In progress* | 21 (9.4%) | 31 (39.7%) |
|  | *Not completed* | 16 (7.2%) | 24 (30.8%) |
|  | ***Total*** | **223 (100%)** | **78 (100%)** |
|  | ***P***^1^ | **<0.001** | |
| **Overall** | *Completed* | 285 (80.7%) | 42 (29.6%) |
|  | *In progress* | 38 (10.8%) | 55 (38.7%) |
|  | *Not completed* | 30 (8.5%) | 45 (31.7%) |
|  | ***Total*** | **353 (100%)** | **142 (100%)** |
|  | ***P***^1^ | **<0.001** | |

^1^ Chi-square test was performed.
