# Supplementary material for: Implementing effective eLearning for scaling up global capacity building: findings from the malnutrition elearning course evaluation in Ghana
Source: Glob Health Action. 2020 Oct 22;13(1):1831794. doi: 10.1080/16549716.2020.1831794 (PMC7595220; doi:10.1080/16549716.2020.1831794)
Supplement: Supplemental Material [file ZGHA_A_1831794_SM8612.docx]

**Supplementary file 7. Differences in knowledge gains (post-pre assessments) by relevance to job/academic progress** (Yes: relevant to job/academic progression; No: not relevant to academic progression)

| **Course completion** | | **Relevant to job / academic progression** | |
| --- | --- | --- | --- |
|  |  | **Group: Yes**^1^ | **Group: No**^2^ |
| Overall | *N* | 365 | 129 |
|  | *Pre, Mean (SD)* | 27.8 (9.9) | 25.1 (9.0) |
|  | *Post, Mean (SD)* | 44.1 (11.9) | 28.9 (9.9) |
|  | *Post – Pre Mean diff (SD)* | 16.3 (11.9) | 3.8 (9.5) |
|  | *95% CI* | (15.1, 17.5) | (2.2, 5.5) |
|  | *P*^3^ | <0.001 | <0.001 |
|  | *Yes – No Mean diff* | 12.5 | |
|  | *95% CI* | (10.2, 14.7) | |
|  | *P*^4^ | <0.001 | |
| Completed | *N* | 291 | 40 |
|  | *Pre, Mean (SD)* | 28.1 (9.8) | 27.4 (8.4) |
|  | *Post, Mean (SD)* | 44.2 (11.9) | 34.2 (8.9) |
|  | *Post – Pre Mean diff (SD)* | 16.1 (11.8) | 6.8 (10.3) |
|  | *95% CI* | (14.7, 17.5) | (3.5, 10.1) |
|  | *P*^3^ | <0.001 | <0.001 |
|  | *Yes – No Mean diff* | 9.3 | |
|  | *95% CI* | (5.4, 13.2) | |
|  | *P*^4^ | <0.001 | |
| In progress | *N* | 39 | 43 |
|  | *Pre, Mean (SD)* | 30.7 (11.8) | 25.2 (10.1) |
|  | *Post, Mean (SD)* | 46.5 (13.8) | 28.1 (10.8) |
|  | *Post – Pre Mean diff (SD)* | 15.8 (13.9) | 2.9 (9.7) |
|  | *95% CI* | (11.3, 20.3) | (-0.04, 5.9) |
|  | *P*^3^ | <0.001 | 0.053 |
|  | *Yes – No Mean diff* | 12.9 | |
|  | *95% CI* | (7.7, 18.1) | |
|  | *P*^4^ | <0.001 | |
| Not completed | *N* | 35 | 46 |
|  | *Pre, Mean (SD)* | 22.6 (6.3) | 23.0 (8.0) |
|  | *Post, Mean (SD)* | 41.0 (8.5) | 25.1 (7.8) |
|  | *Post – Pre Mean diff (SD)* | 18.4 (9.8) | 2.1 (8.1) |
|  | *95% CI* | (15.0, 21.8) | (-0.3, 4.5) |
|  | *P*^3^ | <0.001 | 0.086 |
|  | *Yes – No Mean diff* | 16.3 | |
|  | *95% CI* | (12.3, 20.3) | |
|  | *P*^4^ | <0.001 | |

^1^ MTC group, KNUST and CHK in OD group;

^2^ UHAS in OD, F-CHNTS in ICW and CSUC in MD groups

^3^ Paired *t* test was performed comparing the post assessments scores to the pre assessments scores per course completion and relevance to job/academic progress.

^4^ Independent samples *t* test was performed comparing the post-pre assessments differences between relevance to job/academic progress (Yes – No) by course completion.
